# Direct 3D rotational venography: Insights in optimizing visualization

**DOI:** 10.1177/15910199251329098

**Published:** 2025-03-21

**Authors:** Oleg Shekhtman, Georgios S. Sioutas, Sneha Sai Mannam, Sandeep Kandregula, Joshua S. Catapano, Tina Ehtiati, Jan-Karl Burkhardt, Visish M. Srinivasan

**Affiliations:** 1 189491 Department of Neurosurgery, 14640 Perelman School of Medicine, 6572 University of Pennsylvania, Philadelphia, Pennsylvania, USA; 2 33573 Siemens Healthineers, Malvern, Pennsylvania, USA

**Keywords:** 3D venography, angiography, idiopathic intracranial hypertension, stenosis, navigation

## Abstract

**Introduction:**

Three-dimensional rotational venography (3D-RV) expands on three-dimensional rotational angiography to provide high-quality venous anatomy details, complementing traditional two-dimensional digital subtraction angiography and supporting the diagnosis and treatment of venous pathologies. This article presents a series of patients who underwent advanced 3D-RV for the evaluation of idiopathic intracranial hypertension (IIH).

**Methods:**

In this single-center retrospective case series, we analyzed 13 patients with IIH who underwent direct 3D-RV from June 2023 to May 2024. Access was obtained by placing a 6-Fr or larger guide catheter in the rostral internal jugular vein, with a Zoom 35 microcatheter advanced to the middle third of the superior sagittal sinus. A descriptive analysis was performed based on the demographic and radiation metrics.

**Results:**

Sixteen direct 3D-RV procedures were performed on 13 patients with IIH (mean age 42.06 ± 13.13 years), including 10 females and three males. General anesthesia was administered for interventions (12 cases) and monitored anesthesia care for manometry (four cases). Venous access was obtained via upper extremity veins in 13 cases (81.25%) and the right common femoral vein in three cases (18.75%). Mean fluoroscopy time was 42.0 ± 29.8 min, contrast dose 92.2 ± 34.2 mL, dose area product (DAP) 18.6 ± 10.5 Gy·cm², and air kerma 1.3 ± 0.56 Gy, with a mean procedure time of 71.3 ± 42.0 min. The 3D-RV procedure contributed an additional 1.86 ± 0.6 Gy to DAP and 0.072 ± 0.021 Gy to air kerma, representing an extra 6.26% and 10.59% of the skin dose, respectively. No procedure-related or in-hospital complications occurred.

**Conclusions:**

The 3D-RV procedure is reliable and safe, offering improved accuracy in assessing venous anatomy and stents without significantly impacting procedure time or radiation dose.

## Introduction

Three-dimensional rotational angiography (3D-RA) has become a standard method for assessing arterial vasculature in neurointerventional suites. It is performed using flat-panel fluoroscopy equipment with either one or two rotational acquisitions, each lasting 4–5 s. 3D-RA (also known as 3D-DSA for digital subtraction angiography) utilizes cone-beam computed tomography, offering both angiographic 3D visualization and CT-like multiplanar reformatted (MPR) images.^1,2^ The short scan time facilitates its use in awake patients and allows operators to study neurovasculature in isolation and in relation to surrounding structures.

Although current literature and applications of 3D imaging have primarily focused on arterial vasculature, these techniques could also be beneficial for various venous pathologies. They could enhance understanding of the venous anatomy obtained from traditional 2D digital subtraction angiography (DSA), magnetic resonance venography, and CT venography. Three-dimensional rotational venography (3D-RV) can complement 2D-DSA in evaluating venous anatomy by expanding on the concept of 3D-RA acquisition.

Prior reports demonstrated that 3D-RV can be a powerful adjunct to DSA, showcasing high-quality anatomical details and serving as a navigating tool.^2^ While these initial studies discussed 3D-RV by way of an intraarterial injection with x-ray delay, direct “retrograde” venography has also been described.^3^ We present a retrospective series of patients who underwent advanced 3D-RV examination as part of the workup or treatment for idiopathic intracranial hypertension (IIH).

## Methods

Patients admitted who underwent 3D-RV from 12 June 2023, to 7 May 2024, were identified from our institutional endovascular database. A 6-Fr or larger guide catheter was positioned in the rostral internal jugular vein. A Zoom 35 microcatheter (Imperative Care, Campbell, CA) was positioned in the middle or middle third of the superior sagittal sinus). A microcatheter power injection was performed using the Nemoto Press Duo Elite contrast injector (Nemoto, Berea, OH) with a 6 mL/sec injection rate for a total volume of 26 mL, 0-s injection delay, and 0-s X-ray delay. Importantly, this injection is higher volume than our typical arterial injection of 3 mL/s injection rate for a total volume of 21 mL, due to large vessel size and competitive dilution in the superior sagittal sinus (SSS). Injection pressure limit set at 300 psi and never reached. After stent deployment, a 50% dilution was used for repeated imaging poststenting to enhance visualization in MPR.

Image acquisition was performed using the Siemens Artis Icono biplane system VE21A. The syngo Dyna3D acquisition technique consisted of a 4-s DSA protocol (4-s rotation time, two rotations, 200° angular coverage, 1.50°/frame angulation step, 70 kV, 0.36 µGy/frame). The syngo application software was used for image reconstruction (3D DSA Head, VOI size: Full, Slice matrix: 512 × 512, Kernel type: EE for the subtracted volume).

## Results

Sixteen direct 3D-RV procedures in 13 patients with IIH were included. The mean age of the patients was 42.06 ± 13.13 years, and 10 (76.9%) were female. All but two patients (84.6%) were nonsmokers. Comorbidities included hypertension in three patients, obesity in three, chronic kidney disease in two, dyslipidemia in two, polycystic ovarian syndrome in two, Sjogren disease, iron-deficient anemia, Lyme disease, and myocardial infarction in one patient each. Anesthesia was administered as per our institutional standard: monitored anesthesia care for four manometry procedures and general anesthesia for 12 interventions. For venous access, upper extremity transvenous access was utilized in 13 (81.3%) and right common femoral vein in three (18.75%) cases. 3D-RV was performed in 14 venous stent placement procedures (including two cases of bilateral transverse sinus stenting) and two diagnostic procedures.

The mean fluoroscopy time was 42.0 ± 29.8 min, mean contrast dose was 92.2 ± 34.2 mL, mean dose area product (DAP) was 18.6 ± 10.5 Gy cm², and mean air kerma was 1.3 ± 0.56 Gy. Mean procedure time was 71.3 ± 42.0 min. The 3D-RV procedure added a mean of 1.86 ± 0.6 Gy to DAP and a mean of 0.072 ± 0.021 Gy to air kerma, comprising an extra 6.26% and 10.59% of the skin dose, respectively.

No procedure-related or in-hospital complications were reported. Application of the technique was technically feasible in all cases that were attempted. Although not measured for all cases, in the final three cases the microcatheter phase and acquisition was a minimal time addition to the procedure.

### Illustrative case

**Figure 1.**
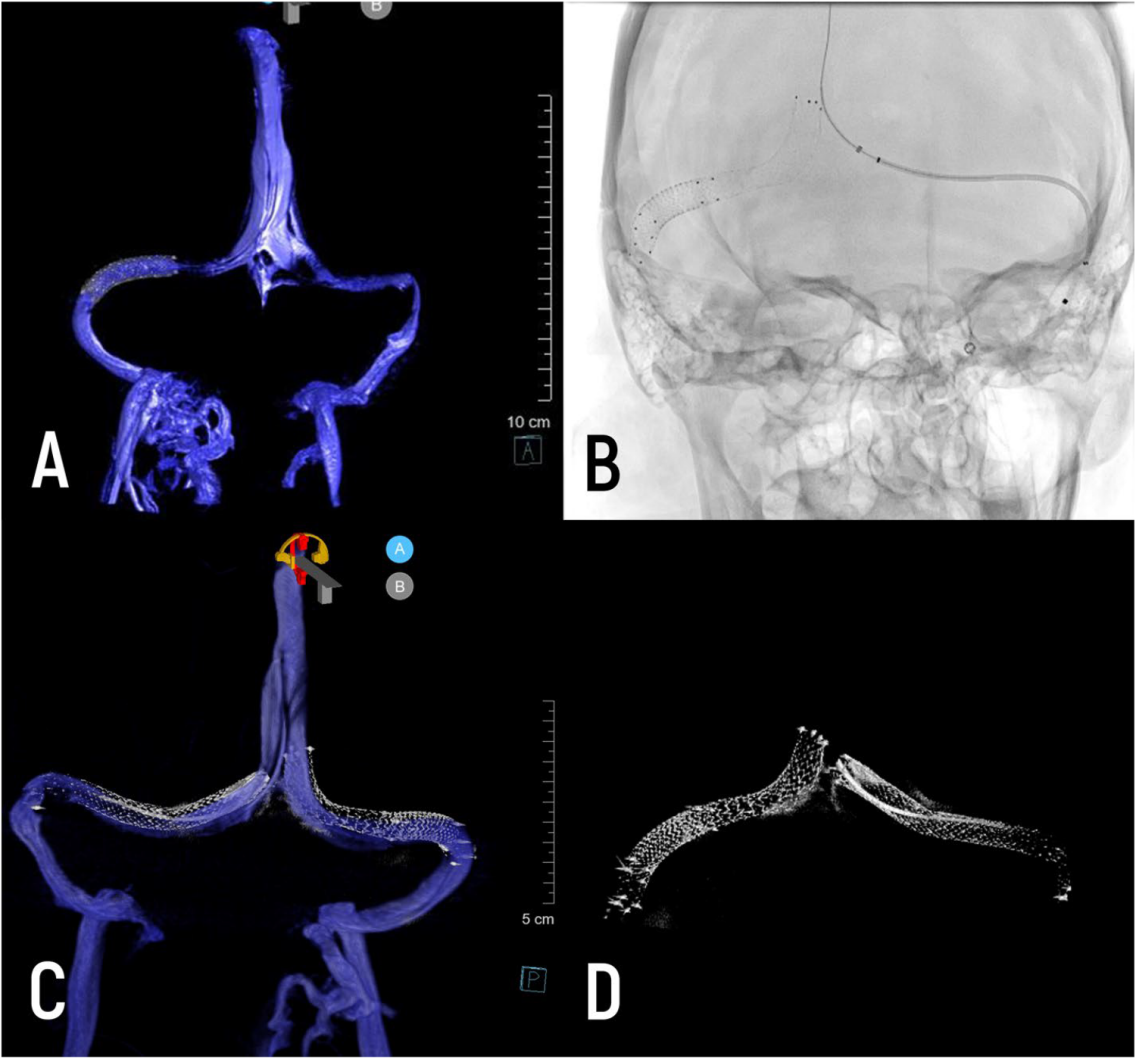
(A) Diagnostic 3D-RV after right transverse sinus stenting; (B) silver stenting system advanced to superior sagittal sinus for left transverse sigmoid stenting; (C, D) bilateral stenting of both transverse sinuses, visualized with 3D-RV and micro-dyn CT.

*History*. A patient in their 30s presented with short recurrent episodes of “vision loss,” eye pressure, pressure headaches, and nausea over the last several months. Ocular fundus exam revealed papilledema. The patient was diagnosed with IIH and underwent diagnostic angiography, venography, and venous manometry. Subsequently, the patient underwent right transverse sinus stenting at an outside hospital. Thirty days later, no significant improvement was observed, prompting the patient's admission for further evaluation and treatment. Venography was repeated (Figure 1) and the stent construct was extended as shown to more aggressively optimize sinus patency on both sides given the initial treatment failure.

3D-RV: three-dimensional rotational venography.

*Procedure*. The procedure was performed under general anesthesia. Using standard transvenous techniques, a stent was placed across the stenotic segment. The stent was recrossed with a Zoom 35 microcatheter and Aristotle 18 microwire. DSA was performed using a Zoom 35 catheter in the middle third of the superior sagittal sinus. This demonstrated improved flow on the right side but persistent venous hypertension and restricted flow. We proceeded to stent the left transverse sinus. Completion angiography was performed using a Zoom 35 catheter in the posterior superior sagittal sinus.

## Discussion

In this technical series, we present our experience with direct 3D-RV as an adjunct to the standard DSA protocol for angiography or venography. The technique was safe and within the range of typical procedures (either diagnostic alone or venous stenting) not using 3D-RV.^4,5^ Acquisition was straightforward and technically feasible in all attempted cases, which included a mix of diagnostic and interventional procedures.

### Evolution of technique

The technique described here is a modification of that described by Young et al. as “retrograde 3D venography.”^3^ In their report of two cases, a 4 Fr catheter was placed in the transverse sinus medial to the stenosis. Injection through this catheter allowed some power injection against flow to opacify the proximal landing zone of the stent and was satisfactory for their application. As experience with venous sinus stenting increases, neurointerventionalists have identified a risk of adjacent segment stenosis of around 11.4%.^6,7^ Experienced neurointerventionalists have overcome this by using longer stent constructs, spanning the vulnerable segments of the venous sinus system.^8^ The direct 3D-RV technique facilitates such constructs, and can be particularly helpful when combined with 3D overlay.

The “retrograde” technique is partially limited by the trackability and stiffness of 4 Fr diagnostic catheters, which may be challenging to navigate across stenoses and too stiff to position in the SSS, especially in conscious patients. Further, they are meant to track over stiffer .035” guidewires, which can increase procedural risk. The large bore microcatheter used here (Zoom 35, ID .035”) is large enough to provide full contrast opacification in the sinus without reaching burst pressure on the power injector, but small and atraumatic enough to navigate into the SSS. Similar large-lumen catheters have been used since this initial series, including the Socrates 38 Aspiration Catheter (Scientia Vascular, Inc., USA) and the AXS Vecta 46 Intermediate Catheter (Stryker Neurovascular, USA). We prefer large bore aspiration microcatheters over diagnostic catheters of similar lumens because of their enhanced trackability and safety profile. Additionally, a significant improvement in image quality is observed when advancing from a 0.027-inch to a 0.035-inch microcatheters.

### Applications for direct 3D-RV

Cerebral angiography and venography, coupled with venous manometry, are now routine procedures for patients with IIH.^9–11^ Imaging during venous stenting also becomes particularly important as this procedure scales with time.^12^ Diagnostic angiography from the arterial side, either with DSA or 3D-RA with x-ray delay, may fail to optimally capture anatomic detail due to flow restriction and washout.^2^ For cerebral venous 3D-DSA, the X-ray delay time can be optimized by using the contrast agent transit time derived from time-enhancement curve analysis.^13^ However, a combination of arterial delay and direct sinus venography may provide a more effective solution. Noninvasive imaging is also prone to error.^14^ Adding 3D-RV to standard DSA is a valuable adjunct, as endoluminal sinus anatomy can vary significantly. Recent studies by McCormick et al. have shown that transverse and sigmoid sinuses frequently carry septations (44%) or blind pouches (42%), which can restrict luminal diameter and alter venous flow.^15^ Altered flow dynamics leading to local stasis or turbulence, as well as blind pouches that may result in thrombi formation in the sinus wall, could potentially explain failed stenting in IIH patients.

High-resolution dural venous sinus imaging could be necessary for a new range of devices, such as endovascular neuroprostheses. It has been recently applied for treatment with the Stentrode (Synchron, Brooklyn, NY) and e-Shunt (Cerevasc, Boston, MA).^16^

## Conclusion

Direct intracranial 3D-RV can provide excellent visualization of the venous sinuses. The anatomic detail provided is particularly useful for the assessment of venous sinus stenosis and in the setting of venous sinus stenting.
